# Orofacial granulomatosis: a questionnaire study among Norwegian dental clinicians

**DOI:** 10.1007/s40368-020-00511-3

**Published:** 2020-02-04

**Authors:** A. B. Skaare, E. S. Hovden, B. B. Herlofson, T. M. Søland

**Affiliations:** 1 Department of Paediatric Dentistry and Behavioural Science, Institute of Clinical Dentistry, Faculty of Dentistry, University of Oslo, Blindern, 1109, 0317 Oslo, Norway; 2 Oral Health Centre of Expertise in Eastern Norway, Oslo, Norway; 3 Department of Oral Surgery and Oral Medicine, Institute of Clinical Dentistry, Faculty of Dentistry, University of Oslo, Oslo, Norway; 4 Institute of Oral Biology, Faculty of Dentistry, University of Oslo, Oslo, Norway; 5 Department of Pathology, Oslo University Hospital, Oslo, Norway

**Keywords:** Orofacial granulomatosis, Oral Crohn disease, Knowledge, Children

## Abstract

**Aims:**

To evaluate awareness on orofacial granulomatosis (OFG) and oral Crohn disease (oral CD) among Norwegian dental clinicians.

**Methods:**

A precoded questionnaire (QuestBack) was sent electronically to dentists and dental hygienists treating children and adolescents in the public dental service (PDS) in Norway. Data on the clinicians’ knowledge of OFG and oral CD related to working experience were analysed by Chi square tests and bivariate logistic regression analyses.

**Results:**

A total of 1097 clinicians were invited to participate, 778 dentists and 319 dental hygienists; 48.2% returned the completed form. Fifty-four percent of the participants had ≥ 10 year experience of clinical practice. Two-thirds (68.4%) of the dentists and all but one of the dental hygienists graduated in Norway. Approximately half of the respondents were aware of OFG (41.2%) and oral CD (57.8%). One-quarter (24.6%) reported that they most likely had seen a patient with OFG and 20.6% with oral CD. Recently graduated dentists (≤ 10 years ago) were more aware of OFG and oral CD than those who graduated > 10 years ago (*p* ≤ 0.001). Regarding dental hygienists, this difference was observed for OFG only (*p* < 0.05). Country of education did not affect the clinicians’ reported knowledge. Approximately 90% would refer a patient suspected of having OFG or oral CD either to a dental specialist or to a physician.

**Conclusion:**

The high prevalence of clinicians observing OFG and oral CD in this study may indicate that OFG and/or oral CD are under-reported and that OFG in particular is more common than hitherto believed. The high frequency of awareness was promising for the benefit of the patients.

## Introduction

Orofacial granulomatosis (OFG) is an uncommon chronic inflammatory disorder involving the mouth, lips, and perioral area. Although OFG is not a life-threatening disease, it often results in increased morbidity due to enlargement of the lips, gingival hyperplasia, and/or painful oral erythema or ulcers (Kolho et al. 2011). The term OFG was first introduced in 1985 to describe all granulomatous inflammatory conditions that are restricted to the face and mouth without signs or symptoms in other organs (Wiesenfeld et al. 1985). Crohn disease (CD) is an inflammatory condition that may affect any part of the gastro-intestinal tract, from the mouth to the anus. It typically affects the distal ileum and colon with symptoms of abdominal pain, diarrhoea, weight loss, and poor appetite. Higher level of pain is associated with reduced quality of life (Claar et al. 2017) and at least one-third of patients have had intestinal surgery within the first 5 years of disease (Hovde and Moum 2012).

The relationship between OFG and CD is still unclear. It is not fully known whether they are different disease entities or the same condition with different involvement of the gastro-intestinal tract (Sanderson et al. 2005; Zbar et al. 2012). It is not possible to distinguish between OFG and oral CD either clinically or histopathologically. However immunological differences are though reported (Freysdottir et al. 2007; Zbar et al. 2012), and recent findings showed that there may be similar phenotypic characteristics despite different genetic characteristics and a different composition of the inflammatory infiltrate (Gale et al. 2014; Gale et al. 2015; Hullah and Escudier 2019).

Although several studies indicate that OFG and CD are two distinct disorders (Challacombe 1997; Sanderson et al. 2005; Grave et al. 2009; Zbar et al. 2012), approximately 40–50% of young patients with OFG will either subsequently develop CD (Leao et al. 2004; Saalman et al. 2009; Rowland et al. 2010; Campbell et al. 2011a) or receive a concomitant CD diagnosis (Lazzerini et al. 2014). Since both OFG and CD often are discovered at young age, OFG may denote an initial presentation of CD or could be a subtype of CD (Saalman et al. 2009; Lazzerini et al. 2014, 2015; Gale et al. 2016). Thus, it is essential to be aware of oral manifestations of OFG as possible initial symptoms of CD.

The aetiology of OFG and CD is still unknown, but studies indicate a multifactorial origin (Tilakaratne et al. 2008; Grave et al. 2009; Al-Hamad et al. 2015; Gale et al. 2015; Hullah and Escudier 2019). In OFG, allergy or hypersensitivity may be a causative factor, since both atopy and contact hypersensitivity are observed in several OFG patients (James et al. 1986; Armstrong et al. 1997; Wray et al. 2000; Fitzpatrick et al. 2011; Campbell et al. 2013; Patel et al. 2013). Studies have shown that a diet free of benzoate and cinnamon have improved the oral symptoms in more than 50% of OFG patients (White et al. 2006; Campbell et al. 2011b). Other dietary products like chocolate (Taibjee et al. 2004; Campbell et al. 2013), food additives (e.g., sorbic acid, glutamate, and carmoisine) (Sweatman et al. 1986; Oliver et al. 1991; Armstrong et al. 1997), perfumes, and flavourings like carvone in spearmint and piperitone in menthol (Patton et al. 1985; Wray et al. 2000) are also shown to be associated with OFG.

In CD, although the aetiology is not known, it probably occurs as an inappropriate immune response to an environmental stimulus in a genetically susceptible person (Hullah and Escudier 2019). Intestinal microbial dysbiosis may also play a role where the gut immune response may be altered by both antibiotic use and dietary changes (Ananthakrishnan 2015; Bernstein 2017).

Being a rare disease, no epidemiological data on OFG exist. Patient characteristics are mainly based on cohorts and case series (Mignogna et al. 2003; Al Johani et al. 2009; Saalman et al. 2009; McCartan et al. 2011; Gale et al. 2015; Haaramo et al. 2017). However, there seems to be an increase in the frequency of OFG in children and young adults in the Western world (Leao et al. 2004; Saalman et al. 2009; Campbell et al. 2011a; Lazzerini et al. 2014) and a prevalence as high as 0.8% has been suggested in the Celtic population (McCartan et al. 2011). Of interest, there is a parallel increase in incidence and prevalence of CD worldwide, in particular in northern Europe and North America (Hovde and Moum 2012; Molodecky et al. 2012; Sjoberg et al. 2014). Approximately one-third of all cases of CD occur in children and adolescents younger than 20 years of age and oral CD has been reported to be present in more than 40% of children at the time of diagnosis of systemic CD (Pittock et al. 2001; Harty et al. 2005).

Since OFG and CD patients often experience increased morbidity, it is best to make an early diagnosis. It is, therefore, important that dental clinicians are familiar with the oral signs and manifestations of OFG and oral CD.

In Norway, all children and adolescents from 3 to 18 years of age are offered free dental care in the public dental service (PDS). Dentists and dental hygienists examine children from 3 years of age on a regular basis and are, thus, in a unique position to make an early diagnosis of oral diseases as well as recognizing manifestations of systemic diseases in the oral cavity. In contrast, physicians usually do not examine the oral cavity. Both OFG and CD are listed among conditions dentists should recognise according to the Scandinavian Fellowship for Oral Pathology and Oral Medicine (SFOPOM) (Kragelund et al. 2012).

The aim of the present article is to report on the awareness of OFG and oral CD among dental clinicians in Norway and to illustrate the oral and perioral features of these conditions.

## Materials and methods

Dental clinicians working in the public dental service (PDS) in 13 out of 19 counties in Norway took part in the study. Between November 2016 and February 2017, a precoded electronic questionnaire (QuestBack) was sent either by electronic mail to the clinicians (in ten counties) or provided as an intranet link (in three counties). The inclusion criteria was clinicians working full time or part time who were treating patients aged 3–20 years.

The Chief Dental Officer in each county encouraged the clinicians to participate in the study and the QuestBack software generated two reminders 2 weeks apart for non-responders. The study was approved by the Norwegian Social Science Data Services (NSD), project number 46517.

**Fig. 1:**
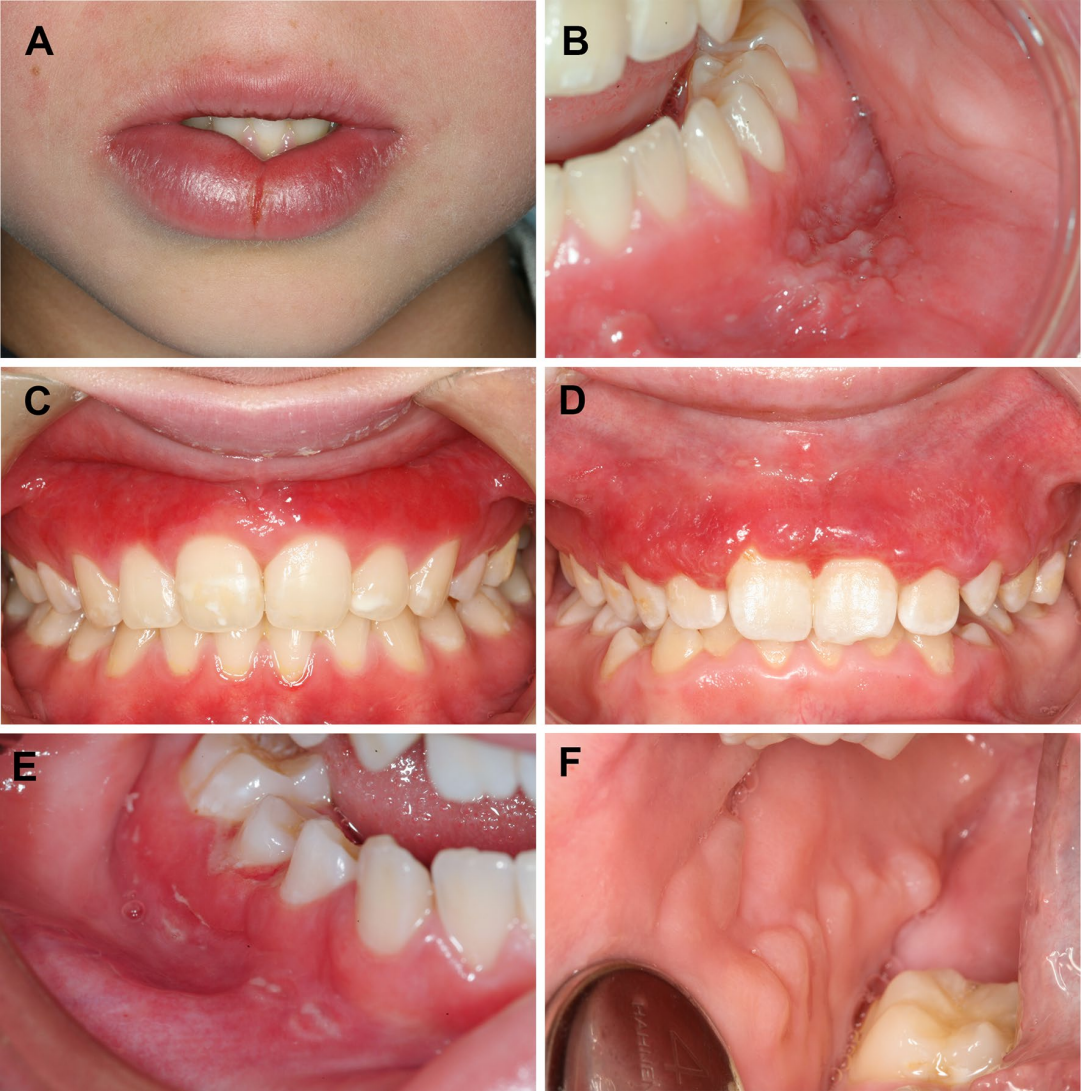
**a–f** Examples of possible oral manifestations in both OFG and oral CD. **a** Lower lip swelling with a midline fissure, **b** vestibular tags lower jaw, **c** gingival erythema upper jaw, **d** oedematous hyperplastic gingiva upper jaw, **e** gingival ulceration buccal sulcus and gingiva region 45, and **f** cobblestoning in right buccal mucosa

In the introduction to the survey, clinicians were informed that patients with OFG and oral CD may present with more than one perioral or oral manifestation and that the symptoms of OFG and oral CD have a cyclical pattern. The questionnaire consisted of 20 precoded and two open-ended questions. Eighteen intra-oral close-up photographs illustrating different manifestations of OFG or oral CD were included, of which six representative pictures of orofacial manifestations are shown in Fig. 1a–f.

The first part of the questionnaire requested demographics of the clinicians (Table 1). Gender, age, number of years in practice, and allocated treatment time with children and adolescents were precoded. Questions on the respondents’ country of education were dichotomized into Norway or abroad.

**Table 1:** Demographics and variables included for the participating clinicians

Characteristics	Dentists (*n* = 373)	Dental hygienists (*n* = 156)
	*n* (%)	*n* (%)
Gender		
Women	287 (76.9)	154 (98.8)
Men	86 (23.1)	2 (1.2)
Age		
21–30 years	71 (19.0)	33 (21.2)
31–40 years	136 (36.5)	32 (20.5)
41–50 years	74 (19.8)	36 (23.0)
> 50 years	92 (24.7)	55 (35.3)
Working experience		
0–5 years	102 (27.3)	36 (23.0)
6–10 years	81 (21.7)	24 (15.4)
11–15 years	47 (12.6)	21 (13.5)
16–20 years	41 (11.0)	26 (16.7)
> 20 years	102 (27.3)	49 (31.4)
Country of education		
Norway	255 (68.4)	155 (99.4)
EU	103 (27.6)	1 (0.6)
Outside EU	15 (4.0)	0 (0.0)
Proportion of full-time equivalent		
20% and below	1 (0.3)	1 (0.6)
21–40%	5 (1.3)	3 (1.9)
41–60%	25 (6.7)	7 (4.5)
61–80%	22 (5.9)	17 (10.9)
81–100%	320 (85.8)	128 (82.1)
Allocated treatment time children		
20% and below	22 (6.9)	1 (0.6)
21–40%	45 (12.1)	7 (4.5)
41–60%	110 (29.5)	21 (13.5)
61–80%	120 (32.2)	55 (35.3)
81–100%	76 (20.4)	72 (46.1)

The main section of the questionnaire included the respondents’ knowledge and awareness of OFG and oral CD, their ability to recognize oral manifestations from clinical photographs, and whether or not they had most likely seen patients with the disorders in their practice.

### Statistics

The data were analysed using SPSS statistical program package (IBM SPSS 25.0, SPSS Inc., Chicago IL, USA). Cross-tabulation with Chi squared (χ2) test was used to identify associations. The ‘number of years in practice’ was dichotomized into more or less than 10 years, and ‘allocated treatment time with children’ and ‘proportion of full-time equivalent’ were dichotomized into more or less than 60% (3 days weekly). Less than 10 years in practice was regarded as recently graduated and the two groups were equal in number. The level of significance was set to 5%. Binary logistic regression analyses were performed to determine associations between the dependent variables ‘knowledge of OFG/oral CD’ and the independent variables ‘working experience’, ‘full-time equivalent’, and ‘allocated treatment time with children’. Odds ratio (OR) with 95% confidence interval (CI) was used to determine the strength of association.

## Results

### Demographics and participants

The selected counties were considered representative of demographics and population, representing clinicians providing care for 75% of the 3–20 years olds living in Norway. A total of 1097 dental clinicians were invited to participate, 778 dentists and 319 dental hygienists.

The completed form was returned by 529 clinicians (48.2%), 373 dentists (47.9%), and 156 dental hygienists (48.9%). The respondents were representative of age (*p* = 0.157) and gender (*p* = 0.213) when compared to all dental clinicians working in the PDS in Norway. Three counties published a link to the questionnaire on their Intranet web site instead of sending electronic mail to the clinicians. This did not affect the representativeness as exclusion of these counties increased the response rate by less than 1%.

Among the participants, 50.9% of the dentists (*n* = 190) and 61.5% of the dental hygienists (*n* = 96) had > 10 years of clinical practice. Two-thirds (68.4%) of the dentists and all but one of the dental hygienists (96.8%) graduated in Norway (Table 1).

### Awareness and knowledge of OFG/oral CD

**Fig. 2:**
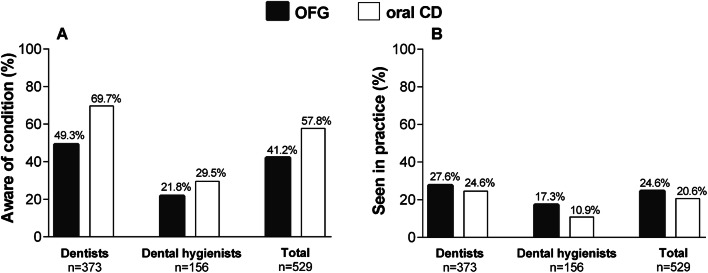
Distribution of knowledge (**a**) and observation (**b**) of OFG and oral CD in practice among clinicians

Of the respondents, 218 (41.2%) were aware of OFG and 130 (24.6%) reported that they had most likely seen a patient with this disorder in the clinic. As shown in Fig. 2, a higher number of clinicians (*n* = 306, 57.8%) were familiar with the term oral CD, but fewer (*n* = 109, 20.6%) reported to have observed these patients in practice.

The association between work experience, proportion of full-time equivalent, proportion of clinical time allocated to children (independent variables), and the knowledge of OFG and oral CD (dependent variable) are presented in Table 2. The most significant finding was that recently, graduated dentists were more likely to report knowledge of OFG and oral CD (OR 3.4 and 2.9, respectively). Recently graduated dental hygienists were also more aware of OFG than dental hygienists graduated more than 10 years ago (*p* = 0.018, OR 2.5), but no significant association was reported on oral CD. There was no association between proportion of full-time equivalent, or proportion of clinical time allocated to children, and awareness of OFG and oral CD (Table 2).

**Table 2:** Binary logistic regression regarding awareness of OFG and oral Crohn (dependent variables) and working experience, clinical working time, and time-allocated children (independent variables)

Aware of condition	Dentists (*n* = 373)							
	OFG				Oral CD			
	*n* (%)	*p* value	OR	95% CI	*n* (%)	*p *value	OR	95% CI
Working experience								
≤ 10 years (*n* = 183)	119 (65.0)	0.000*	3.5	(2.2; 5.3)*	148 (80.9)	0.000*	2.9	(1.8; 4.7)*
> 10 years (*n* = 190)	65 (34.2)				112 (58.9)			
Proportion of full-time equivalent								
≤ 60% (*n* = 31)	12 (38.7)	0.217	0.8	(0.4; 1.8)	20 (64.5)	0.511	0.9	(0.4; 2.2)
> 60% (*n* = 342)	172 (50.3)				240 (70.2)			
Proportion of clinical time-allocated children								
≤ 60% (*n* = 177)	76 (42.9)	0.532	0.8	(0.5; 1.2)	116 (65.5)	0.323	0.9	(0.6; 1.4)
> 60% (*n* = 196)	106 (54.1)				141 (71.9)			

Aware of condition	Dental hygienists (*n* = 156)							
	OFG				Oral CD			
	*n* (%)	*p* value	OR	95% CI	*n* (%)	*p *value	OR	95% CI
Working experience								
≤ 10 years (*n* = 60)	19 (31.7)	0.018*	2.5	(1.1; 5.4)*	22 (36.7)	0.12	1.7	(0.8; 3.5)
> 10 years (*n* = 96)	15 (15.6)				24 (25.0)			
Proportion of full-time equivalent								
≤ 60% (*n* = 11)	1 (9.1)	0.289	0.4	(0.4; 2.8)	2 (18.2)	0.393	0.5	(0.1; 2.5)
> 60% (*n* = 145)	33 (22.8)				44 (30.3)			
Proportion of clinical time-allocated children								
≤ 60% (*n* = 29)	4 (13.8)	0.247	0.5	(0.2; 1.7)	7 (24.1)	0.484	0.7	(0.3; 1.9)
> 60% (*n* = 127)	30 (23.6)				39 (30.7)			

*Statistically significant difference

Regarding observations of OFG and oral CD patients in the clinic, no association with working experience, full-time equivalent, or allocated treatment time with children in either of the professions was noted.

The dentists who graduated from Norway did not differ in knowledge compared to those who graduated abroad. Awareness regarding OFG was reported to be 50.2% and 47.5%, respectively. All but one dental hygienists graduated in Norway. If the clinicians were encountered with a patient with suspected OFG/oral CD, approximately 90% would refer the patient either to a dental specialist or to a physician.

## Discussion

The literature shows that OFG may have its onset in childhood and that an unpredictable subgroup of children with OFG may develop CD. Both OFG and CD are intermittent with steady and acute phases.

In OFG and oral CD, a predominant clinical finding is lip swelling followed by intra-oral features like hyperplastic gingivitis and cobblestoning, oral ulcers, and mucosal tags (Pittock et al. 2001; Harty et al. 2005; Al Johani et al. 2010; Campbell et al. 2011a; McCartan et al. 2011; Haaramo et al. 2017). However, it is reported that the overall discomfort, as well as the duration of symptoms and problems with appearance, are more severe in OFG than in oral CD patients (Gale et al. 2015; Miest et al. 2016). The treatment is challenging and often unsatisfactory, but the importance of early diagnosis must not be underestimated as oral manifestations are often resolved in children when treated for CD (Hussey et al. 2011).

Studies of the knowledge, awareness, and experience of general dental practitioners and dental hygienists concerning OFG and oral CD are lacking. Since both conditions are an increasing problem, especially in the Western world, it is important to evaluate the knowledge of OFG and CD among dental clinicians. The aim of the present survey was to focus on these two conditions paying a special attention to OFG, as this can be painful, disfiguring, and possibly be the first sign of a systemic disease (CD) in the individual. There is no referral protocol for children suspected of OFG or oral CD in Norway, but nearly all the clinicians in the present study reported that they would refer these patients.

In the present study, dentists and dental hygienists working in the PDS were included. A strength of the study was that it included dental clinicians in PDS; all children and adolescents in Norway are offered regular free dental care, and 97.6% of 1–18 years old were enrolled in the service in 2017 (https://www.ssb.no/statbank/table/11985/tableViewLayout1/). This makes it possible for dental clinicians to identify and diagnose individuals with OFG and oral CD at an early stage and age. Due to the nature of the disease which has an intermittent course and symptoms, it is essential that clinicians know the spectrum of possible clinical manifestations. As dental hygienists are the first-line clinicians in the PDS, it was very important to include this profession in the survey.

The response rate was not optimal. However, web surveys usually have modest response and the present study is in line with the literature (Blumenberg and Barros 2018). The response rates in surveys have been steadily falling in recent years due to increased questionnaire-based research activity. This may cause an increased burden on clinicians, and thus, it is more difficult to obtain response rates previously accepted as normal. The current study was questionnaire-based and participation was voluntary. Thus, selection bias related to personal interests of clinicians may have occurred.

Approximately 50% of the respondents were aware of both OFG and oral CD. A statistically significant difference in this knowledge was seen according to the number of years in clinical practice. Recently graduated dentists (≤ 10 years) were more aware of OFG and oral CD than those graduated more than 10 years ago. Among the dental hygienists, the same difference was observed regarding OFG only. This difference between the professions is not easily explained. The terms OFG/oral CD are often used interchangeably, despite being two distinct disorders, and the distinction between the two disorders may have been less clear for dental hygienists. The general high awareness among all of the clinicians may be explained by a stronger emphasis on the disorders in the students’ curricula in recent years, the fact that the conditions are more commonly seen today than in the past or a combination of these factors. The high number of clinicians reporting to have seen patients with oral CD may also be a reflection on the increasing incidence of CD in the Western world in recent years (Lehtinen et al. 2011; Hovde and Moum 2012; Molodecky et al. 2012; Sjoberg et al. 2014). However, neither ‘working experience’, ‘proportion of full-time equivalent’, or ‘allocated treatment time to children’ were associated with the clinicians’ answers of observations in practice.

No epidemiological data on OFG exist. This may be due to the challenge of getting reliable information on this intermittent and rare disorder. Here, a surprisingly high percentage of dental clinicians (24.6%) reported to have seen OFG in their clinical practice. This potentially suggests an increase in incidence of OFG, as is the case for CD (Saalman et al. 2009; McCartan et al. 2011). The clinicians were informed that patients with OFG and oral CD might present with more than one perioral or oral manifestation. The 18 photos presented in the questionnaire showed different patients focusing on separate oral manifestations but with only one clinical characteristic in each (examples in Fig. 1 where all but one have confirmed Crohn disease, 1–13 years after OFG diagnosis). This may have been misleading, since each manifestation in itself is not specific for OFG or oral CD. For example, many clinicians have probably observed facial erythema and angular cheilitis in their practice, as atopic dermatitis is increasing in developing countries affecting up to 20% of children and adolescents (Mei-Yen Yong and Tay 2017). Thus, dental clinicians may have reported seeing OFG patients in practice with atopy or eczema only and not a concomitant OFG. Other common findings in OFG are swollen lips, intra-oral enlargement of the gingiva (granulomatous gingivitis), erythema, ulcerations, vestibular tags, or a cobblestone appearance of the buccal mucosa (McCartan et al. 2011; Gale et al. 2015). These findings may be misdiagnosed as a hypersensitivity or allergic reaction, gingivitis, or aphthous ulcers as the clinical presentation of OFG and oral CD are not pathognomonic (Campbell et al. 2011a). Thus, there is a degree of uncertainty in relation to the final data.

It is essential that dental clinicians are able to distinguish the different clinical manifestations as painless gingival hyperplasia may be misdiagnosed as plaque-induced gingivitis (Harty et al. 2005) or gingivitis induced by mouth breathing (Mignogna et al. 2003; Al-Hamad et al. 2015). Lip swellings and enlargement of gingiva are challenging as the disfigurement may result in psychological morbidity (Al Johani et al. 2010; Al-Hamad et al. 2015).

The clinicians’ observations were not found to be influenced by country of graduation, as there was no difference between dentists who qualified in or outside Norway. This finding reflects that the focus on OFG and CD in Norway is in line with the dental education in other countries. One-quarter of the clinicians reported that they most likely had seen a patient with OFG and one-fifth had most likely seen a patient with oral CD. Even though fewer clinicians reported that they had seen oral CD in their practice, more than half were familiar with the term. These figures may indicate that the conditions are under-reported.

The treatment of OFG is challenging and often unsatisfactory and the importance of early diagnosis must not be underestimated. Children under the age of 16 with oral symptoms were shown to be more likely to develop CD than if the oral presentation was after that age (Campbell et al. 2011a). Clinicians should be aware of the highly variable nature of OFG to provide early diagnosis as these patients are often seen by many health professionals prior to receiving a diagnosis, which can be frustrating for the patient.

## Conclusion

The survey suggests that OFG and oral CD could be more common than hitherto believed. The high frequency of awareness was promising for the benefit of the patients who may get an early diagnosis and tailored treatment based on a correct diagnosis.
